# Characterization and evaluation of a recombinant multiepitope peptide antigen MAG in the serological diagnosis of *Toxoplasma gondii* infection in pigs

**DOI:** 10.1186/s13071-021-04917-w

**Published:** 2021-08-17

**Authors:** Yongle Song, Yongjuan Zhao, Ke Pan, Bang Shen, Rui Fang, Min Hu, Junlong Zhao, Yanqin Zhou

**Affiliations:** 1 Key Laboratory Preventive Veterinary of Hubei Province, College of Veterinary Medicine, Huazhong Agricultural University, Wuhan, 430070 Hubei People’s Republic of China; 2 State Key Laboratory of Agricultural Microbiology, Huazhong Agricultural University, Wuhan, 430070 Hubei People’s Republic of China

**Keywords:** *Toxoplasma gondii*, Pig, Synthetic multiepitope antigen, Indirect ELISA, Serological detection

## Abstract

**Background:**

Toxoplasmosis caused by *Toxoplasma gondii* is a serious disease threatening human and animal health. People can be infected with *T. gondii* by ingesting raw pork contaminated with cysts or oocysts. Serological test is a sensitive and specific method usually used for large-scale diagnosis of *T. gondii* infection in humans and animals (such as pigs). Commercial pig *Toxoplasma* antibody ELISA diagnostic kits are expensive, which limits their use; moreover, the wide antigen composition used in these diagnostic kits is still unclear and difficult to standardize. The multiepitope peptide antigen is a novel diagnostic marker, and it has potential to be developed into more accurate and inexpensive diagnostic kits.

**Methods:**

The synthetic multiepitope antigen (MAG) cDNA encoding a protein with epitopes from five *T. gondii*-dominant antigens (SAG1, GRA1, ROP2, GRA4, and MIC3) was designed, synthesized, and expressed in *Escherichia coli* BL21 (DE3) strain. The recombinant protein was detected through western blot with pig anti-*T. gondii*-positive and -negative serum, and then IgG enzyme-linked immunosorbent assay (ELISA) named MAG-ELISA was designed. The MAG-ELISA was evaluated in terms of specificity, sensitivity, and stability. The MAG-ELISA was also compared with a commercial PrioCHECK^®^ *Toxoplasma* Ab porcine ELISA (PrioCHECK ELISA). Finally, the trend of pig anti-*T. gondii* IgG levels after artificial infection with RH tachyzoites was evaluated using MAG-ELISA and two other ELISA methods (rMIC3-ELISA and PrioCHECK ELISA).

**Results:**

MAG antigen could be specifically recognized by pig anti-*T. gondii*-positive but not -negative serum. MAG-ELISA showed high diagnostic performance in terms of specificity (88.6%) and sensitivity (79.1%). MAG-ELISA could be used for detecting anti-*T. gondii* IgG in the early stage of *T. gondii* infection in pigs (at least 7 days after artificial infection).

**Conclusions:**

Our results suggest that MAG antigen can be applied to specifically recognize anti-*T. gondii* IgG in pig, and MAG-ELISA has the potential for large-scale screening tests of *T. gondii* infection in pig farms and intensive industries.

**Graphical abstract:**

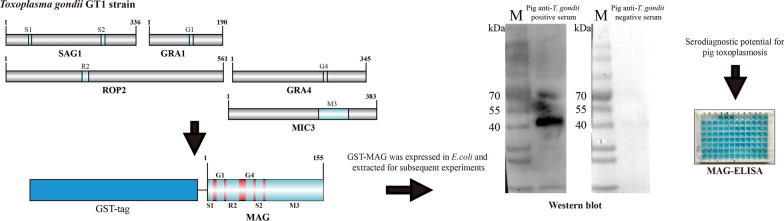

**Supplementary Information:**

The online version contains supplementary material available at 10.1186/s13071-021-04917-w.

## Background

*Toxoplasma gondii* is an apicomplexan intracellular protozoan parasite, and it can infect all warm-blooded vertebrates, including humans and domestic animals [1]. This parasite threatens human and animal health especially for pregnant and in immunocompromised individuals [2, 3]. Humans can be infected with *T. gondii* by ingesting food and raw pork contaminated with cysts or oocysts [4, 5]. Pork is the main meat source in many countries, such as China. Many epidemiological investigations have shown that pig farms and intensive industries have high prevalence and parasite load by PCR detection and serological test, but the detection of *T. gondii* in pigs is usually not taken seriously in pig farms and intensive industries because of the expense of diagnosis and high error rate [6–8]. Therefore, the development of simple, inexpensive, and sensitive diagnostic tests for *T. gondii* detection in pigs is crucial to reduce the risk of toxoplasmosis in humans and pigs.

The diagnostic approach to toxoplasmosis has been constantly evolving, including traditional techniques (e.g., immunology and imaging tolls) and many emerging molecular techniques. The etiological diagnosis of toxoplasmosis is relatively time-consuming since it involves the isolation of numerous disease materials and requires considerable skills to obtain reliable results. Thus, it is impossible to apply etiological diagnosis to large-scale clinical tests in pig farms and intensive industries. Imaging diagnosis is mainly applied to cerebral and ocular toxoplasmosis using large medical equipment, including computed tomography (CT), magnetic resonance imaging (MRI), nuclear imaging, and ultrasonography (US), but imaging diagnostic results may not be reliable and require expert interpretation [9]. Molecular techniques are widely applied to the epidemiological survey and clinical diagnosis of toxoplasmosis because of their accuracy and sensitivity [10]. The molecular technique used for toxoplasmosis diagnosis is a high-sensitivity nucleic acid detection method for parasites in biological samples, and it overcomes the limitations of the serological tests; in addition, molecular techniques mainly include PCR, nested PCR, real-time PCR, loop-mediated isothermal amplification (LAMP), and recombinase polymerase amplification (RPA) assay [11–13]. However, parasite nucleic acid detection involving DNA extraction tends to be expensive, and it is only accessible in the laboratory. Immunological detection is common method to determine the immune status of the host by examining the change patterns of several different specific antibodies (IgA, IgM, IgG and IgE) after *T. gondii* infection [1, 14]. The common immunological method of toxoplasmosis diagnosis includes enzyme-linked immunosorbent assays (ELISA), modified agglutination test (MAT), and others [15–17].

ELISA is a serological detection that can be easily performed on a large scale, and many commercial kits are available to detect specific immunoglobulins (Igs) after *T. gondii* infection. The solid-phase antigen used for ELISA includes crude tachyzoite antigen, *Escherichia coli* recombinant antigen, and chimeric peptide antigen. Although *Toxoplasma* lysate antigen (TLA) has high sensitivity and specificity levels in ELISA, there are problems with TLA such as false-positive results, standardization difficulty, unclear antigen composition, and complex and expensive TLA preparation [18, 19]. It is impossible to detect all serologically positive individuals by using one or several *Escherichia coli* recombinant antigens, because the expression patterns of antigen genes from different *T. gondii* strains vary during different infection stages [20]. Synthetic multiepitope antigen, also known as chimeric antigen, is a new generation of recombinant product for ELISA, and it contains multiple immunoreactive epitopes from several dominant antigens of *T. gondii*. Multiepitope antigens have been widely used for toxoplasmosis diagnosis; for example, synthetic multiepitope antigens are applied to detect anti-*T. gondii* IgG and IgM, and AMA1-SAG2-GRA1-ROP1 chimeric antigens are used to detect specific antibodies of human and mouse in early and chronic *T. gondii* infection [21]. Chimeric antigen technology has been developed for the serological diagnosis of *Trypanosoma cruzi* infection caused by another protozoan parasite, cutaneous anthrax caused by *Bacillus anthracis*, and others [22–24]. However, few studies have been conducted to evaluate chimeric antigens for serodiagnosis of *T. gondii* in pigs and to design an ELISA kit using synthetic antigens for the large-scale diagnosis of toxoplasmosis in pig farms and intensive industries.

Many *T. gondii* proteins are mainly secreted outside through three specific organelles (rhoptry, dense granule, and microneme), some of which could well activate the host immune system. *T. gondii* surface antigen 1 (SAG1), as a highly immunogenic protein, is mainly distributed on the tachyzoite surface by glycosyl-phosphatidylinositol anchoring [25, 26]. Dense granule proteins 1 and 4 (GRA1 and GRA4) secreted by *T. gondii* have good antigenicity [27–30]. Rhoptry protein 2 (ROP2) belonging to ROP2-protein family is expressed in three stages (tachyzoites, bradyzoites, and sporozoites) of *T. gondii*'s life cycle, and this protein induces a strong antibody response in mice and humans [31, 32]. Microneme protein 3 (MIC3), as an adhesion molecule expressed in *T. gondii*, could be recognized by anti-*T. gondii*-positive serum. Mice immunized with recombinant pseudorabies viruses expressing MIC3 can produce high levels of anti-*T. gondii* IgG to provide effective protection against *T. gondii* challenge in BALB/c mouse model [33]. Although these antigens (SAG1, GRA1, ROP2, GRA4, and MIC3) have been well documented to stimulate host immunity, little work has been done to determine whether the chimeric antigen with their T and/or B cell epitopes is a good diagnostic marker for toxoplasmosis in pig farms and intensive industries.

To develop an efficient and inexpensive ELISA kit for toxoplasmosis diagnosis in pigs, our study tried to synthesize a multiepitope antigen (MAG) cDNA from five *T. gondii*-dominant antigens (SAG1, GRA1, ROP2, GRA4 and MIC3) and evaluate the possibility of applying the chimeric protein MAG to the ELISA diagnosis of *T. gondii* infection in pigs.

## Methods

### MAG recombinant chimeric antigen

**Fig. 1:**
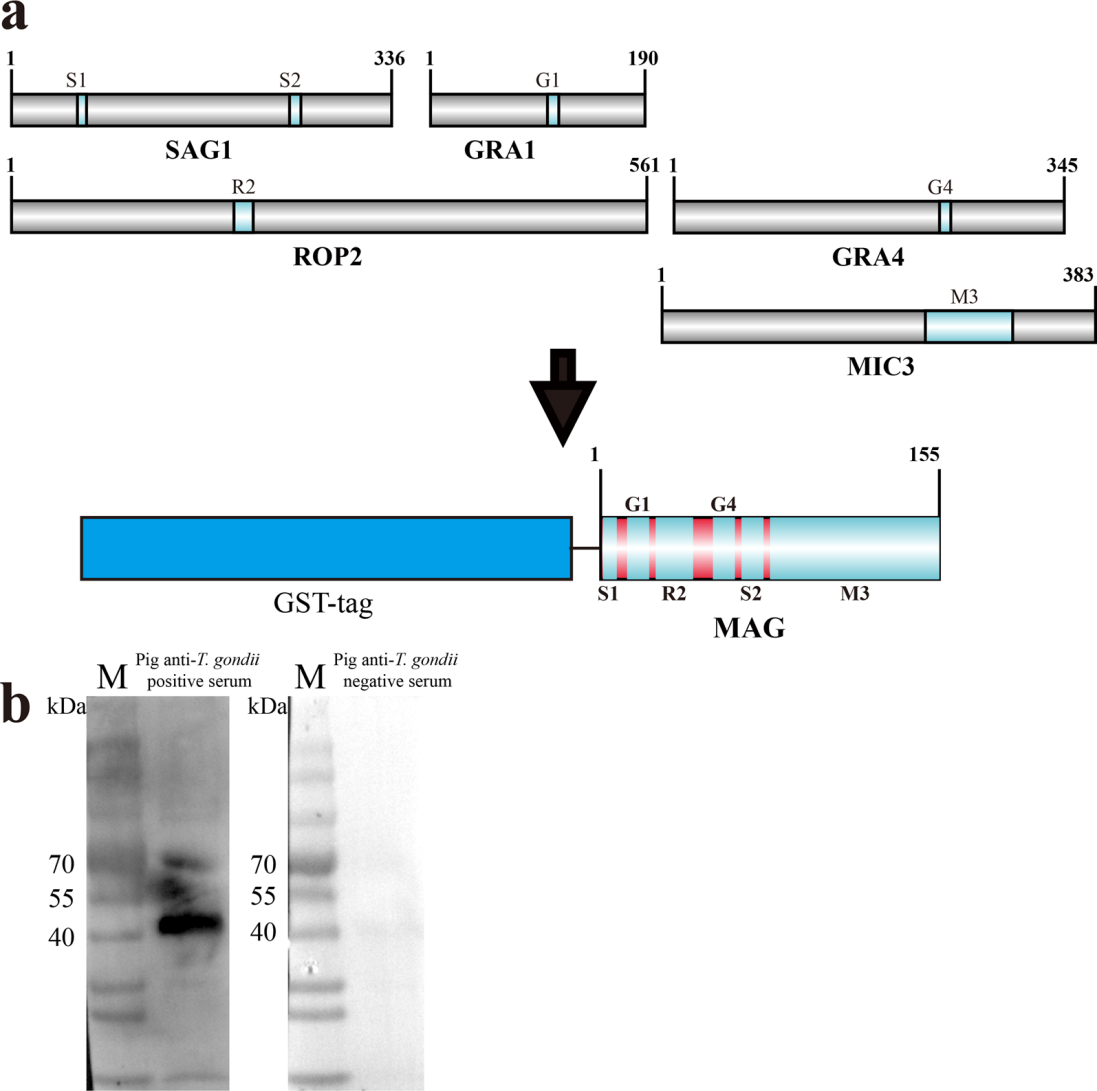
MAG construction and antigen identification. **a** Construction of chimeric antigen MAG. **b** Western blot to identify MAG antigenicity

The epitope was selected according to two parts. The T/B-cell epitope of each antigen was predicted by Immune Epitope Database (IEDB) with default parameters, and the antigen was also analyzed by DNAstar Lasergene 11 with default parameters. MAG was designed by concatenating peptides containing T and/or B cell epitopes (Table 1, Fig. 1a). The MAG cDNA used in the indirect ELISA was synthesized (TSINGKE Biological Technology), and then the synthetic cDNA fragment was cloned into the pGEX-KG expression vector and expressed in *Escherichia coli* BL21(DE3) strain. MAG recombinant protein fused with glutathione S-transferase (GST) was induced with IPTG (isopropyl β-d-1-thiogalactopyranoside) and then purified by glutathione-based affinity chromatography (GE Healthcare Life Sciences, USA). Finally, the purified MAG antigens were quantified by enhanced bicinchoninic acid (BCA) protein assay kit (Beyotime Biotechnology, China).

**Table 1:** Sources of chimeric antigen MAG

Protein	Epitope	Strain	Sequence	Position
SAG1	S1	GT1	TCPDKKSTA	59–67
	S2	GT1	ILPKLTENPWQ	246–256
GRA1	G1	GT1	DTMKSMQRDED	104–114
ROP2	R2	GT1	PGDVVIEELFNRIPETSV	197–214
GRA4	G4	GT1	SGLTGVKDSSS	235–245
MIC3	M3	GT1	KRTGCHAFRE…SCKCDNGYSG	233–310

### Western immunoblot analysis

The purified MAG protein was separated by SDS-PAGE to identify the protein molecular weight through protein molecular weight marker (ThermoFisher scientific) and analyzed by western blot. The Immobilon-PSQ PVDF membrane (0.2 μm pore size, Millipore, USA) onto which purified protein was transferred was incubated with pig anti-*T. gondii*-positive or -negative serum and then detected with horseradish peroxidase (HRP)-conjugated rabbit anti-swine IgG (H+L) (Frdbio Bioscience & Technology, China).

### Pig artificial infection with tachyzoite

“Chang xin” binary miscellaneous commodity pigs (any gender, weight of 15–20 kg) bred by the pig farm of Huazhong Agricultural University were used to perform RH (type I strain) tachyzoite artificial infection by intraperitoneal injection. Four pigs were randomly assigned into two groups with two pigs in the infected group and two in the control group. Two pigs in the infected group were intraperitoneally injected with 5 million tachyzoites for *T. gondii* infection. The two pigs in the control group were intraperitoneally injected with an equal volume of sterile 0.9% NaCl; 2 ml of serum samples was collected from the infected and control group on days 0, 2, 4, 7, 14, 21, 28, 35, 42, and 49. The serum sample at day 3 before *T. gondii* infection was also collected for subsequent detection.

### Optimization of ELISA procedure

ELISA was performed as described previously [20]. The 96-well flat-bottom microtiter plates (BIOFIL, China) were coated overnight at 4 °C with 100 µl per well of coating buffer (25 mM carbonate buffer, pH 9.6) containing MAG-purified antigen. The 96-well plate was washed three times with wash buffer (phosphate-buffered saline (PBS) with 0.05% Tween 20). Blocking buffer (bovine serum albumin (BSA) in PBS) was added, and then the plate was incubated for a period of time at 37 °C. After three rewashes as described above, 100 µl of pig serum diluted with serum dilution buffer was added to each well, and the plates were incubated for a period of time at 37 °C. After being washed as described above, 100 µl of HRP conjugated recombinant Protein A/G (ThermoFisher Scientific) diluted in blocking buffer was added to each well, and the plates were incubated for a period of time at 37 °C. After being washed as described above, color was developed for a period of time at 37 °C in a dark room after the addition of 100 µl per well of the substrate solution containing 3,3ʹ,5,5ʹ-tetramethylbenzidine (TMB, Sigma-Aldrich, USA) and H_2_O_2_. Finally, the OD630 value was measured with a Bio-Tek ELx-800 microplate reader (BioTek Instruments, USA). All the tests were performed in duplicate wells.

To obtain the optimal dilution ratio of MAG-coated antigen and pig serum, the cross-titration was performed at serial dilution ratios of MAG antigen (1:100, 1:200, 1:400, 1:800, 1:1600, and 1:3200), *T. gondii*-positive serum, and negative pig serum samples (1:20, 1:40, 1:80, 1:160, 1:320, and 1:640). The OD630 was measured, and the ratio of positive/negative serum (P/N) was calculated at different dilution ratios. The dilution ratios of MAG antigen and pig serum with the maximum value of P/N were designated to be the optimal dilution ratio.

The optimal blocking buffer concentration (0.5%, 1.0%, 1.5%, and 2.0% BSA) and the optimal blocking time (30, 40, 50, and 60 min) were determined as described above. The optimal serum dilution buffer (0.1% BSA, 0.5% BSA, and PBS containing 0.05% Tween 20) was determined by the maximum value of P/N. The optimal coating time of pig serum (30, 40, 50, and 60 min) was determined by the maximum value of P/N under the above optimal conditions. The optimal dilution ratio (1:3000, 1:4000, 1:5000, 1:6000, 1:7000 and 1:8000) and the optimal reaction time (30, 40, 50 and 60 min) of HRP-conjugated recombinant Protein A/G were also determined by cross-titration. The optimal reaction time of TMB substrate solution (5, 7.5, 10, and 12.5 min) was determined by the maximum value of P/N. The cutoff value of MAG-ELISA was determined referring to the OD630 mean value of 25 *T. gondii*-negative pig sera under optimal conditions (cutoff = mean + 3 SD).

### Evaluation of MAG-ELISA

The cross-reactivity of MAG-ELISA was assessed by the diagnosis of five common pig virus sera [swine fever virus (SFV), porcine reproductive and respiratory disease virus (PRRSV), pseudorabies virus (PrV), porcine circovirus (PCV), and foot-and-mouth disease virus (FMDV)] and the control group including pig anti-*T. gondii*-positive and -negative sera. The lowest detection limit of MAG-ELISA was determined by measuring OD630 at serial dilution concentrations of pig anti-*T. gondii*-positive sera. The coefficient of variation (CV) of repeated tests within batches and between batches was calculated and confirmed respectively using six samples (one anti-*T. gondii*-positive and five -negative pig sera). The stability was tested by the destruction experiments in which MAG-coated plates were placed at 37 °C for 12, 24, 36, 48, 60, and 72 h, respectively. The 209 pig serum samples were tested by MAG-ELISA and a commercial PrioCHECK^®^ *Toxoplasma* Ab porcine ELISA (Prionics, Switzerland), respectively. The OD450 cutoff value of PrioCHECK ELISA was calculated according to the manual. The sensitivity and specificity of MAG-ELISA versus PrioCHECK ELISA were assessed according to two ELISA results.

### Procedure of rMIC3-ELISA

The rMIC3-ELISA was performed in a previously reported ELISA method for detecting pig toxoplasmosis [34, 35]. Entire recombinant *T. gondii* microneme protein 3 (rMIC3) fused with GST tag was expressed and purified as coated antigen. The 96-well plates were coated with rMIC3 at 3.40 µg/ml, and pig serum samples were diluted at 1:160. The cutoff value of rMIC3-ELISA was defined as 0.40. The OD630 value was measured as described above.

## Results

### Characterization of recombinant multiepitope antigen and optimization of MAG-ELISA procedure

Our study selected six epitopes from five reported *T. gondii*-dominant antigens (SAG1, GRA1, ROP2, GRA4, and MIC3) and designed a new chimeric recombinant multiepitope antigen (MAG) by concatenating peptides containing T and/or B cell epitopes (Table 1, Fig. 1a). MAG was synthesized and cloned into pGEX-KG vector for prokaryotic expression. The MAG protein with GST tag (43.3KD) was successfully expressed in *Escherichia coli* BL21 (DE3) and purified through glutathione-based affinity chromatography. Western blot results showed the MAG protein was recognized specifically by pig anti-*T. gondii*-positive serum but not by -negative serum (Fig. 1b).

**Fig. 2:**
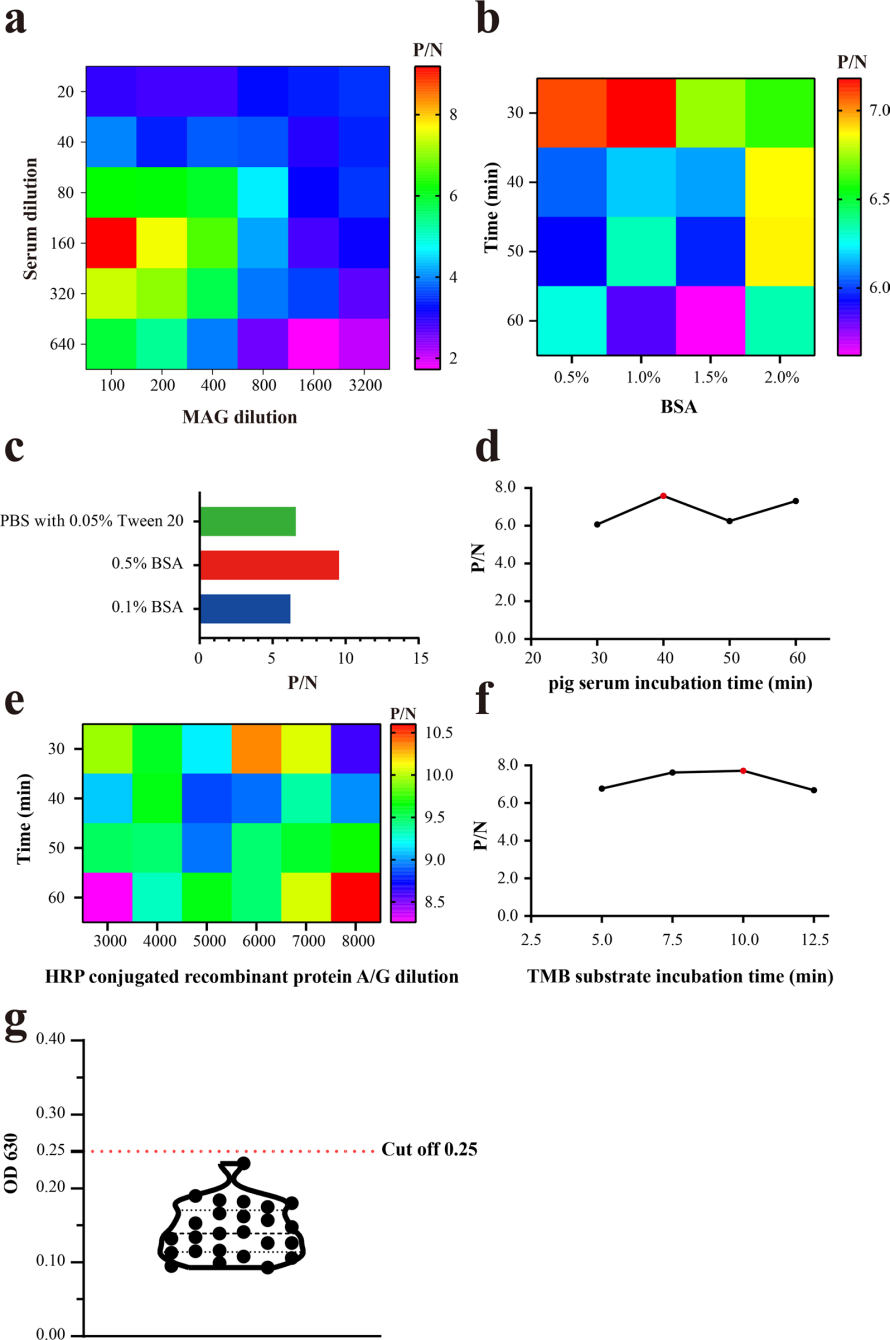
Optimization of MAG-ELISA. **a** Optimal dilution ratio of MAG antigen and pig serum. **b** Blocking buffer optimization of BSA concentration and incubation time. **c** Optimization of pig serum dilution buffer. **d** Optimization of pig serum incubation time. **e** Optimization of HRP conjugated recombinant Protein A/G dilution ratio and incubation time. **f** Optimization of TMB substrate incubation time. **g** Cutoff value of pig anti-*Toxoplasma gondii*-negative serum (cutoff = mean + 3 SD)

To improve the performance of MAG-ELISA, we attempted to optimize the procedures related to MAG antigen, pig serum, buffer, secondary antibody, and ELISA substrate, and others. The cross-titration showed that the optimal dilution ratio of MAG-coated antigen was 1:100 (0.91 µg/well) and that of pig serum was 1:160 (Fig. 2a). The maximum P/N was obtained when a MAG-coated plate was incubated with 1.0% BSA for 30 min under the optimal conditions as described above (Fig. 2b). We also found 0.5% BSA was the optimal serum dilution buffer compared to 0.1% BSA and PBS containing 0.05% Tween 20 (Fig. 2c). The optimal pig serum incubation time was determined to be 40 min (Fig. 2d). HRP-conjugated recombinant Protein A/G exhibited optimal performance when the plates were incubated at 1:8000 for 60 min (Fig. 2e). The optimal color development was obtained after TMB substrate was incubated for 10 min (Fig. 2f). The cutoff value of MAG-ELISA was set as 0.25 after testing 25 *T. gondii* negative pig sera under the optimal conditions described above (Fig. 2g). The subsequent MAG-ELISA was performed according to the above-mentioned optimal procedures.

### Evaluation of MAG-ELISA

**Fig. 3:**
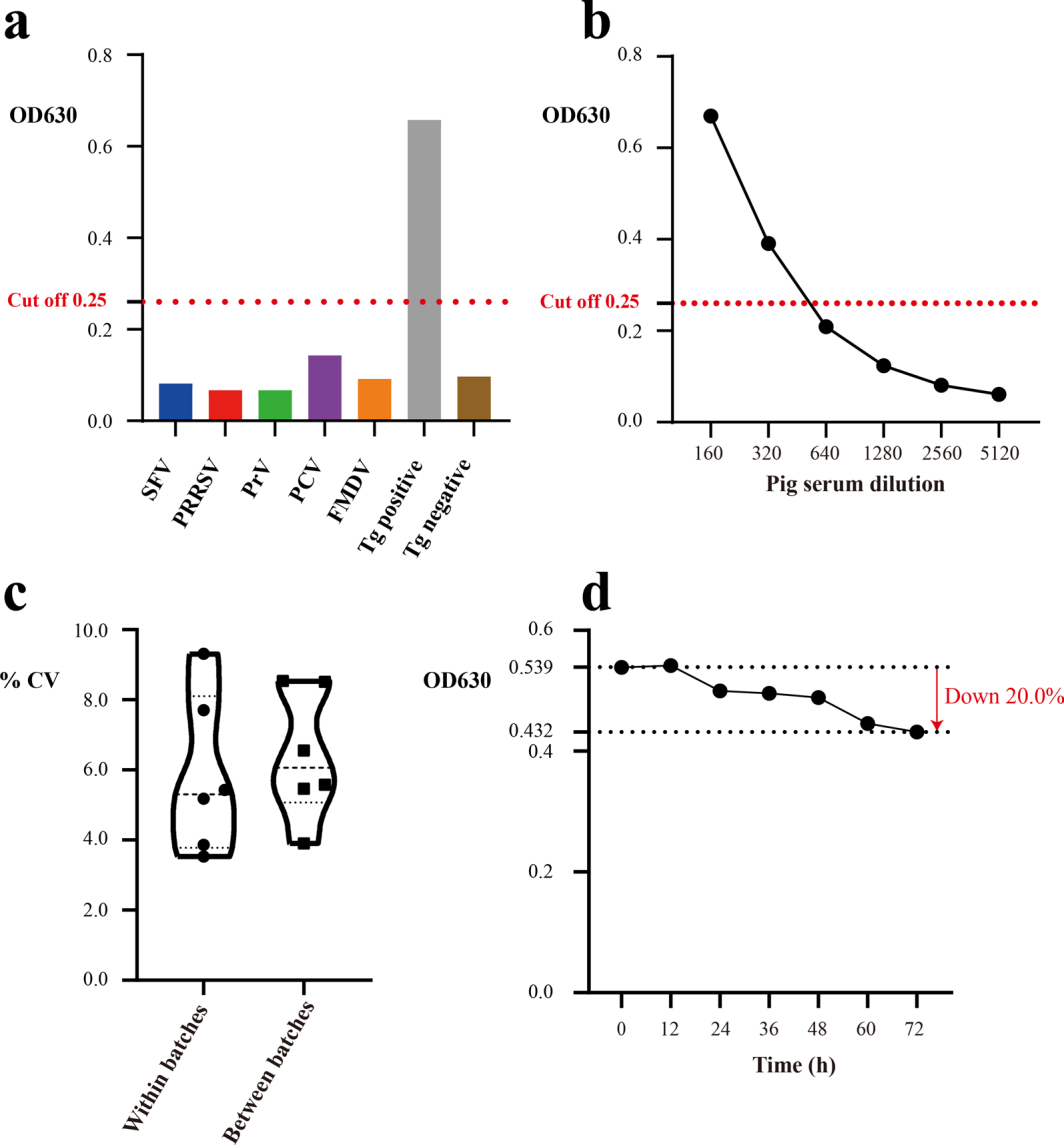
Evaluation of MAG-ELISA. **a** Cross-reactivity of MAG-ELISA was assessed by the diagnosis of five common pig viruses (SFV, PRRSV, PrV, PCV, and FMDV). **b** Lowest detection limit of MAG-ELISA was determined by serial dilutions of pig anti-*Toxoplasma gondii*-positive serum. **c** CV of repeated tests within batches and between batches. **d** The MAG-ELISA stability was tested by the destruction experiment at 37 °C for different times

To evaluate the feasibility of our MAG-ELISA, we also examined its specificity, sensitivity, stability, and others. The OD630 values of the five common pig viruses (SFV, PRRSV, PrV, PCV, and FMDV) were significantly lower than the cutoff value of MAG-ELISA (Fig. 3a), indicating our ELISA did not exhibit obvious cross-reaction with these five common pig viruses, which confirmed the good specificity of MAG-ELISA. By measuring OD630 at serial dilutions of *T. gondii*-positive pig serum, we found that pig anti-*T. gondii*-positive serum was still determined as positive even at the dilution ratio of 1:320, indicating the sensitivity of MAG-ELISA was adequate (Fig. 3b). The CV of repeated tests within batches and between batches was all < 10.0%, suggesting the good stability of our MAG-ELISA (Fig. 3c). After placing MAG-coated plates at 37 °C for 72 h, the OD630 only dropped by < 20.0% (Fig. 3d). We further compared MAG-ELISA with PrioCHECK ELISA by testing 209 pig serum samples simultaneously. The sensitivity of MAG-ELISA was 79.1%, specificity was 88.6%, positive predictive value (PPV) was 82.9%, and negative predictive value (NPV) was 85.8% (Table 2, Additional file 1: Tables S1 and S2).

**Table 2:** Comparison of MAG-ELISA and PrioCHECK ELISA to detect *Toxoplasma gondii* infection

	PrioCHECK ELISA		Total
	Positive	Negative	
MAG-ELISA			
Positive	68	14	82
Negative	18	109	127
Total	86	123	209

### MAG-ELISA detection of artificial pig infection with type I tachyzoite

**Fig. 4:**
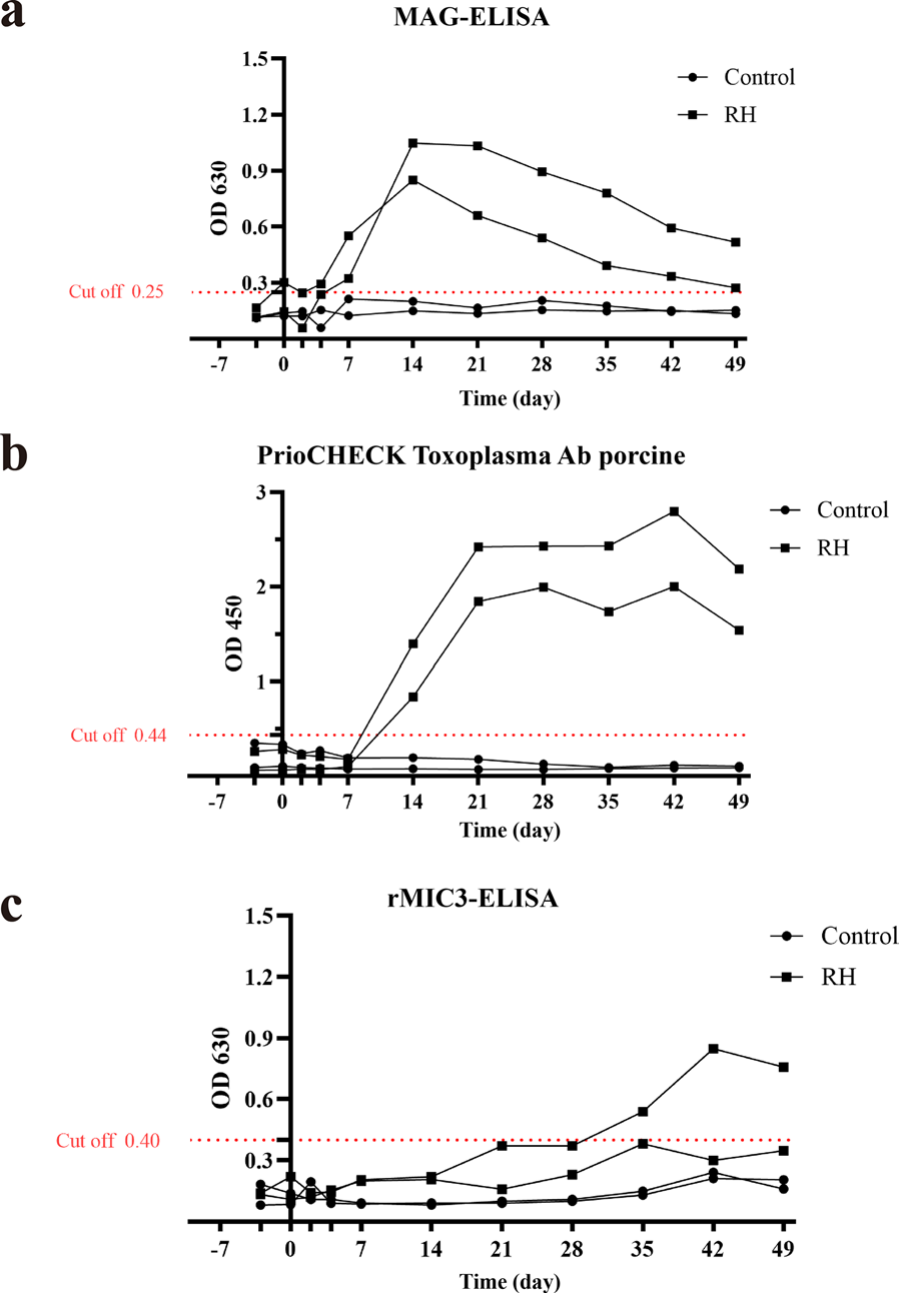
Trend of pig anti-*Toxoplasma gondii* IgG level after artificial infection with RH tachyzoites in MAG-ELISA and two other ELISA methods

MAG-ELISA and two other ELISA methods (rMIC3-ELISA and PrioCHECK ELISA) were applied to detect pig anti-*T. gondii* IgG level after artificial infection with RH tachyzoites. We collected pig serum samples from infected group and control group at days 0, 2, 4, 7, 14, 21, 28, 35, 42, and 49 after infection and at day 3 before infection, respectively. MAG-ELISA detected anti-*T. gondii*-positive IgG in the early stage of pig infection (at least 7 days), which was earlier than the results obtained by rMIC3-ELISA and PrioCHECK ELISA at days 35 and 14, respectively (Fig. 4). Two weeks after artificial infection, our MAG-ELISA indicated that the overall level of anti-*T. gondii* IgG had gradually decreased, but rMIC3-ELISA and PrioCHECK ELISA showed that the level of anti-*T. gondii* IgG reached its maximum value after day 35 post-infection. Therefore, MAG-ELISA could be applied for early detection of *T. gondii* infection in pig farms and intensive industries.

## Discussion

Humans can be infected by *T. gondii* by digesting raw or uncooked meats (such as pork). Infection with *T. gondii* can lead to reproductive disorders in sows (such as miscarriage, stillbirths, and weak fetuses). Poor meat quality threatens human health and causes serious economic losses to the livestock industry. *T. gondii* can survive for a long time in many pig tissues such as heart, lungs, and brain in the form of cysts. Pork is considered to be one of the main sources of human toxoplasmosis [4, 36]. A high prevalence of *T. gondii* is found in pig farms, especially organic farms and intensive pig industries [37, 38]. Therefore, it is necessary to develop a highly sensitive, specific, and inexpensive kit for the large-scale diagnosis of pig toxoplasmosis in farms and intensive industries.

ELISA is a common method for the large-scale detection of infectious disease mainly caused by pathogenic bacteria, viruses, and parasites. The specificity and sensitivity of ELISA depend mainly on the coated antigen. All the common coated antigens such as TLA and recombinant antigen have their own limitations. For example, the composition of TLA is poorly understood, and TLA preparation procedure is complex and can be infectious for the operator. Although recombinant protein antigen of *T. gondii* is very useful for serodiagnosis of toxoplasmosis, one recombinant antigen can detect only one type of anti-*T. gondii* IgG, and the diagnosis result may be affected by the expression level of antigen gene [39]. Thus, the application of these antigens in ELISA may cause a high error rate in large-scale detection. The chimeric antigen encoded by synthetic multiepitope antigen cDNA is a new generation of recombinant products, and it has competitive advantages since it contains more immunoreactive epitopes from several dominant antigens of *T. gondii* than conventional recombinant antigen. Chimeric antigen with the epitope from no more than three *T. gondii* antigens used in ELISA has been reported to perform well in toxoplasmosis diagnosis [19]. To further improve the performance of ELISA, our MAG-ELISA used a chimeric antigen containing six epitopes of T and B cells from five *T. gondii*-dominant antigens (SAG1, GRA1, ROP2, GRA4, and MIC3). SAG1 is only highly expressed in acute infection, but GRA1, ROP2, ROP4, and MIC3 are expressed in acute and chronic *T. gondii* infection, endowing MAG antigen with the potential to detect pig anti-*T. gondii* IgG in two different infection periods. Our results showed that MAG-ELISA possessed high specificity, sensitivity, and repeatability. The test of 209 pig serum samples indicated that the sensitivity of MAG-ELISA was 79.1%, specificity was 88.6%, PPV was 82.9%, and NPV was 85.8% (Table 2, Additional file 1: Tables S1 and S2). The seroprevalence of *T. gondii* infection between MAG-ELISA (39.2%) and PrioCHECK ELISA (41.1%) showed little difference, indicating that MAG-ELISA is valuable for large-scale screening of *T. gondii* infection in pig farms. The diversity of test results between MAG-ELISA and PrioCHECK ELISA was mainly related to the weak positive and negative samples in PrioCHECK ELISA. The accuracy of serological testing methods is always limited by the level of antibodies detected in the serum samples. The antigen used in commercial PrioCHECK ELISA was formalin-fixed cell culture-derived tachyzoite antigen, but the antigen used in MAG-ELISA was several peptides from five *T. gondii*-dominant antigens (SAG1, GRA1, ROP2, GRA4, and MIC3). The more types of antigens in commercial kits, the greater the potential for the detection of *T. gondii* infection in pigs, but for PrioCHECK ELISA antigens with unclear components, false positives are inevitable. The low total IgG level in MAG-ELISA might be attributed to the inconsistence in the levels of anti-*T. gondii* individual IgGs against these six epitopes at different infection stages. Furthermore, false-positive results of the PrioCHECK ELISA also might be caused by some sample test results being close to the cutoff value because of the unclear component of TLA antigen. It is possible to improve the sensitivity and specificity of the recombinant multiepitope peptide antigen in the serological diagnosis of *T. gondii* infection by using a large number of peptides that are highly expressed during acute and chronic infection.

We further compared the obtained levels of anti-*T. gondii* IgGs after artificial infection with RH tachyzoites through three different ELISAs (MAG-ELISA, rMIC3-ELISA, and PrioCHECK ELISA). Our result showed that MAG-ELISA could detect *T. gondii* infection at day 7 after infection, which was earlier than the other two ELISA methods. However, the level of anti-*T. gondii* IgG obtained through MAG-ELISA was inconsistent with those through rMIC3-ELISA and PrioCHECK (Fig. 4). The reasons for the above-mentioned inconsistency might be as follows. First, pig anti-*T. gondii* IgGs against the epitopes of SAG1, GRA1, ROP2, GRA4, and MIC3, respectively, had inconsistent levels after artificial RH infection. Second, the IgG trend in MAG-ELISA could represent the overall level of several anti-*T. gondii* IgGs. Thus, our MAG-ELISA should be more reliable than the ELISA using only one recombinant antigen. However, the MAG-ELISA could detect anti-*T. gondii* IgG only within 1 to 7 weeks after artificial infection with a detection time span shorter than that of the other two ELISA methods, which might be due to the low expression of the antigen genes corresponding to some epitopes. Previous study also indicated that antibody responses to GRA1 and MIC3 were very weak or even absent 6 weeks post infection [40].

MAG could specifically recognize pig anti-*T. gondii* IgG; thus, MAG-ELISA has potential for diagnosing *Toxoplasma* infection in pigs. MAG-ELISA could help improve the accuracy and reduce the cost of toxoplasmosis diagnosis in pig farms and intensive industries. MAG-ELISA can monitor the trend of change in anti-*T. gondii* IgG after artificial pig infection, increasing our knowledge about *T. gondii* infection in pigs. Further work is also needed to optimize the performance of the MAG-ELISA and verify its diagnostic effect in detecting acute and chronic *T. gondii* infection in pigs and other animals (such as pets).

## Conclusions

We report that MAG, as a synthetic multiepitope antigen, can recognize pig anti-*T. gondii*-positive but not -negative serum. MAG-ELISA had good diagnostic performance in terms of specificity and sensitivity compared with a commercial PrioCHECK ELISA. MAG-ELISA has potential for use in large-scale diagnosis of *T. gondii* infection in pig farms and intensive industries.

## Supplementary Information

**Additional file 1: Table S1.** Diagnosis results of 209 pig serum samples by MAG-ELISA. **Table S2.** Diagnosis results of 209 pig serum samples by PrioCHECK ELISA.

## Data Availability

All relevant data are within the paper and additional file.

## Abbreviations

MAG: Synthetic multiepitope antigen
ELISA: Enzyme-linked immunosorbent assay
PrioCHECK ELISA: PrioCHECK^®^ *Toxoplasma* Ab porcine ELISA
TLA: *Toxoplasma* lysate antigen
SAG1: *T. gondii* surface antigen 1
GRA1: Dense granule protein 1
GRA4: Dense granule protein 4
ROP2: Rhoptry protein 2
MIC3: Microneme protein 3
BCA: Bicinchoninic acid
HRP: Horseradish peroxidase
IPTG: Isopropyl β-d-1-thiogalactopyranoside
GST: Glutathione S-transferase
PBS: Phosphate buffered saline
BSA: Bovine serum albumin
TMB: 3,3ʹ,5,5ʹ-Tetramethylbenzidine
P/N: Positive/negative serum
SFV: Swine fever virus
PRRSV: Porcine reproductive and respiratory disease virus
PrV: Pseudorabies virus
PCV: Porcine circovirus
FMDV: Foot-and-mouth disease virus
CV: Coefficient of variation
rMIC3: Recombinant entire *T. gondii* microneme protein 3
PPV: Positive predictive value
NPV: Negative predictive value
